# Procedure for Handling and Storage of *Onchocerca volvulus* Microfilariae Obtained from Skin Snips for Downstream Genetic Work

**DOI:** 10.3390/tropicalmed8090445

**Published:** 2023-09-12

**Authors:** Shannon M. Hedtke, Anusha Kode, Tony O. Ukety, Jöel L. Mande, Germain M. Abhafule, Anuarite A. Raciu, Claude B. Uvon, Stephen R. Jada, An Hotterbeekx, Joseph Nelson Siewe Fodjo, Makedonka Mitreva, Wilson Sebit, Robert Colebunders, Warwick N. Grant, Annette C. Kuesel

**Affiliations:** 1 Department of Environment and Genetics, La Trobe University, Bundoora, VIC 3086, Australia; a.kode@latrobe.edu.au (A.K.); w.grant@latrobe.edu.au (W.N.G.); 2 Centre de Recherche en Maladies Tropicales (CRMT), Bunia P.O. Box 143, Democratic Republic of the Congo; tony.ukety@gmail.com (T.O.U.); joel.mande6@gmail.com (J.L.M.); abhafule@gmail.com (G.M.A.); anuariteraciu@gmail.com (A.A.R.); claudeuvon3@gmail.com (C.B.U.); 3 Amref South Sudan, Juba P.O. Box 30125, South Sudan; stephen.jada@amref.org; 4 Global Health Institute, University of Antwerp, 2610 Antwerp, Belgium; an.hotterbeekx@uantwerpen.be (A.H.); josephnelson.siewefodjo@uantwerpen.be (J.N.S.F.); robert.colebunders@uantwerpen.be (R.C.); 5 Department of Medicine, Washington University in St. Louis and McDonnell Genome Institute, St. Louis, MO 63108, USA; mmitreva@wustl.edu; 6 National Public Health Laboratory, Juba P.O. Box 88, South Sudan; wilsonladu0@gmail.com; 7 UNICEF/UNDP/World Bank/World Health Organization Special Programme for Research and Training in Tropical Diseases (TDR), World Health Organization, CH-1211 Geneva, Switzerland; kuesela@who.int

**Keywords:** onchocerciasis, microfilariae, drug trials, epidemiological studies, genetic analysis

## Abstract

WHO and endemic countries target elimination of transmission of *Onchocerca volvulus*, the parasite causing onchocerciasis. Population genetic analysis of *O. volvulus* may provide data to improve the evidence base for decisions on when, where, and for how long to deploy which interventions and post-intervention surveillance to achieve elimination. Development of necessary methods and tools requires parasites suitable for genetic analysis. Based on our experience with microfilariae obtained from different collaborators, we developed a microfilariae transfer procedure for large-scale studies in the Democratic Republic of Congo (DRC) comparing safety and efficacy of ivermectin, the mainstay of current onchocerciasis elimination strategies, and moxidectin, a new drug. This procedure is designed to increase the percentage of microfilariae in skin snips suitable for genetic analysis, improve assignment to metadata, and minimize time and materials needed by the researchers collecting the microfilariae. Among 664 microfilariae from South Sudan, 35.7% and 39.5% failed the mitochondrial and nuclear qPCR assay. Among the 576 microfilariae from DRC, 16.0% and 16.7% failed these assays, respectively. This difference may not only be related to the microfilariae transfer procedure but also to other factors, notably the ethanol concentration in the tubes in which microfilariae were stored (64% vs. ≥75%).

## 1. Introduction

Onchocerciasis (or river blindness) is a filarial nematode disease that predominantly impacts people in sub-Saharan Africa. *Onchocerca volvulus* is transmitted through the bites of blackflies of the genus *Simulium*. A fraction of the adult *O. volvulus* (length of males 3–5 cm, length of females 30–80 cm) resides in subcutaneous nodules which can be excised by minor surgery, while the majority are inaccessible deep in the body. *Onchocerca volvulus* microfilariae (mf, length 220–360 µm) are primarily present in the skin [1,2].

For studies of the efficacy and/or safety of drugs, the standard method to quantify the level of skin mf is to take skin snips, incubate them for several hours in physiological saline until the mf have emerged, and count the mf microscopically [3]. This method is also used for epidemiological studies that require knowledge of the level of skin mf, usually with a lower number of skin snips than obtained during drug efficacy trials (e.g., [4,5,6]). Methods for detecting the presence of *O. volvulus* in skin snips via quantitative polymerase chain reaction (qPCR) and loop-mediated isothermal amplification (LAMP) have been developed but are not widely used [7,8].

The World Health Organization (WHO) and endemic countries, in partnership with other stakeholders, are targeting elimination of transmission of *O. volvulus*, using primarily annual mass drug administration of ivermectin (MDAi) [9,10,11]. Elimination of parasite transmission has been WHO-verified in most of the small, isolated endemic areas in the Americas, where less than 0.6 million people were estimated to live across 11 different endemic areas, and where biannual MDAi, complemented in hyperendemic villages with quarterly MDAi, was feasible [12,13]. Elimination faces much bigger challenges in sub-Saharan Africa where endemic areas are vast [14,15]. While parasite transmission may have been eliminated in some areas in Africa or may be close to elimination [11,16,17,18], WHO estimated that in 2021 around 243.7 million people in the WHO African Region lived in areas requiring interventions to achieve elimination of *O. volvulus* transmission, 0.9 million in the WHO Eastern Mediterranean Region (Sudan, Yemen), and 0.035 million in the American Region (Brazil, Venezuela) [13].

National programs in Africa face a number of challenges for achieving elimination of *O. volvulus* transmission including, but not limited to, the remoteness of endemic communities, the size of the endemic areas including hyperendemic areas [14,15], transmission between endemic areas and countries caused by movement of infected people and infected/infective vectors [19], and the lack of objective criteria to delineate the geographical areas that constitute different transmission zones (foci). Delineation of transmission zones is important for two reasons: (1) to ensure that interventions are implemented where needed, and (2) because transmission zones are also the ‘geographical unit’ for the evaluations recommended by WHO to assess whether parasite transmission has been eliminated [9]. 

Our goal is to develop tools for national program managers that will help them address some of these challenges, including tools for discrimination of parasites from different areas and delineation of transmission zones [19,20,21], assessment of the risks for recrudescence if interventions are stopped in some geographic areas while transmission is ongoing in other parts of the transmission zone [19], monitoring the decline in the number of reproductively active parasites [22], and monitoring the prevalence of parasites responding sub-optimally to ivermectin [23]. To support development of these tools, we are exploiting the information available in the parasite and vector genomes [24]. 

The SARS-CoV2 pandemic has increased awareness of the potential that analysis of the genome of infectious agents has for surveillance and control of infectious diseases. Utilization of genomics for tropical diseases lags far behind. Proposals to develop genetics/genomics-based tools for malaria control go back 10 years [25] but widespread use has not yet been achieved. Use cases for such tools include identification of transmission foci and connectivity between parasite populations, characterizing the origins and dynamics of outbreaks, monitoring import of malaria cases and their contribution to local transmission, and monitoring of parasite susceptibility to antimalarial drugs [26,27]. Similar use cases can be made for the control and elimination of filarial diseases, as recently reviewed for onchocerciasis and lymphatic filariasis [24].

One prerequisite for development of genome-based tools is availability of well-preserved parasites for DNA extraction and subsequent sequencing. Well-preserved parasites, e.g., in biobanks supporting researchers world-wide [28], may also support research for other tools for onchocerciasis elimination programs, such as development of sufficiently sensitive and specific new diagnostics to detect patent *O. volvulus* infection [29,30] or development of vaccines [31,32,33]. 

We have received *O. volvulus* mf from skin snips taken from participants from multiple studies that were all preserved, labelled, and shipped in different ways. We observed that vials provided to us might not include any parasites or substantially fewer than the accompanying information indicated (i.e., inadequate transfer of mf into tubes for preservation), that leakage of ethanol during transport of parafilm-wrapped tubes stored in plastic bags resulted in blurring or even erasure of handwritten labeling (i.e., inadequate labeling and storage), that sample identifiers written on the tops of parafilm-wrapped tubes were removed by unwrapping of the parafilm, and that the proportion of mf that yielded DNA of sufficient quality for sequencing varied substantially. 

Based on this experience we have developed a procedure for transfer and storage of skin mf for two ongoing studies in the Democratic Republic of the Congo (DRC) which compare the safety of a single dose of moxidectin or ivermectin (study MDGH-MOX-3002) and the safety and efficacy of three annual and five biannual treatments with moxidectin or ivermectin (study MDGH-MOX-3001) (https://mox4oncho-multimox.net/resources (accessed on 7 April 2023)). This procedure simplifies the process for transferring mf that emerged from skin snips into storage media, for provision of metadata (e.g., location of collection, time point of collection within a study, microscopically determined mf count in the skin snip), and for linking the metadata to tubes with mf. It also facilitates leakage-free transport from the research institutions that collect the samples to the laboratories that analyze them. We share our experiences with mf samples transferred with this procedure from the ongoing studies in DRC and without this procedure from an epidemiological study on onchocerciasis-associated epilepsy in South Sudan [34,35] for downstream DNA-based research (as in [22]).

## 2. Experimental Design

### 2.1. Microfilariae Transfer for Genetic Analysis from a 2018 Epidemiological Study in the Maridi Health District of South Sudan without the Newly Developed Procedure

In the study that did not use our procedure [34,35,36], two skin snips (1–2 mg each) were obtained from each study participant and incubated for 24 h in physiological saline at ambient temperature. Once mf had emerged into physiological saline, ~400–500 μL was placed on a microscope slide with a cover slip, and mf counted under a 10× ocular lens. The remaining mf were transferred together with 100 μL of the saline with an automatic pipette into flat-capped Eppendorf tubes filled with 1000 μL 70% ethanol (final ethanol concentration ~64%). Each tube was labelled with the participant and study time identifier using a solvent resistant pen and wrapped in parafilm. The tubes were packaged in plastic bags according to international requirements for shipping biological samples and sent to La Trobe University (Victoria, Australia) via international courier. The metadata (participant code, geographic origin, time point within the study, number of mf in each skin snip) were provided via a spreadsheet emailed to collaborators.

### 2.2. Microfilariae Transfer for Genetic Analysis from the Ongoing Trials in DRC with the New Procedures

Four skin snips (1–2 mg each; from the right and left iliac crest and right and left calf) were obtained from each person screened for the ongoing clinical trials in DRC (MDGH-MOX-3002 and MDGH-MOX-3001) and incubated in physiological saline. Mf that had emerged were transferred for shipping via international courier to La Trobe University as per the procedure we designed for these studies, described in detail below.

## 3. Overview of the Procedure

**Figure 1:**
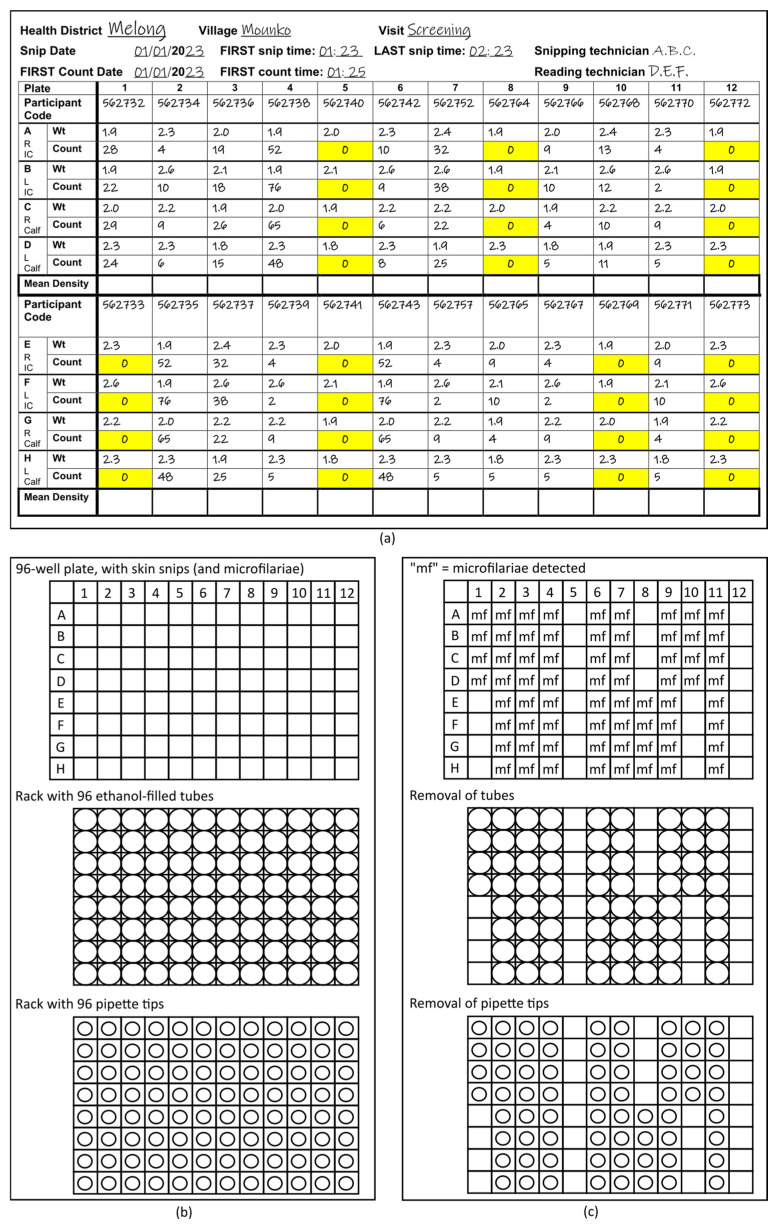
Schematic of 96-format microfilariae transfer procedure. (**a**) Sample Source Record Form with right (R) and left (L) iliac crest (IC) and calf skin snip weight (Wt), microfilariae count, geographic origin, and study time point (and information required for assessing compliance with the standard operating procedure for obtaining skin microfilariae counts). Zero counts have been highlighted in yellow for this schematic. (**b**) Aligned 96-well plate, 96-format rack with ethanol-filled tubes, and 96-format rack with pipette tips. (**c**) Aligned 96-well plate, 96-format rack with ethanol-filled tubes, and 96-format rack with pipette tips after removal of ethanol tubes and pipettes corresponding to positions of wells without microfilariae.

1. After counting, the procedure for transfer and storage of mf is designed to reduce researcher workload and fatigue-related human error as follows:
  - Use of the “96-format” (12 columns and 8 rows) throughout the process. For the incubation of the mf in 0.9% saline before counting, the Source Record Forms (Figure 1a) on which the parasitologists record the number of mf counted microscopically in each well (Supplemental File: Source Record Forms for four or two skin snips per participant) and the racks with ethanol-filled tubes into which the mf are transferred. The 96-format is also taken into consideration by use of racks with 96 pipette tips and either 8-channel or 12-channel pipettes.
  - Labelling of the 96-format racks, rather than individual tubes with mf, using positional information to associate wells in the 96-well plates in which the skin snips are incubated, corresponding metadata on the 96-format Source Record Form and the individual tubes in the 96-tube racks into which the mf are transferred from the 96-well plates (Figure 1b,c).
  - Each rack used has a unique identifier or barcode. That barcode is entered into a label printer and printed in duplicate. Both printed labels are attached to the rack: one will remain on the rack, the other will be attached to the Source Record Form that includes the metadata for the mf to unambiguously link the samples that will be transferred to tubes in the rack with the Source Record Form.
2. To reduce the probability of sample loss through the use of the following:
  - Screw-top tubes, which reduce evaporation and accidental opening during handling and shipping.
  - Racks with a lockable lid, which prevent tubes from falling out of place during transport. This is critical for a procedure that eliminates the workload and potential errors associated with labelling many tubes individually by associating tube position with metadata.
3. To preserve resources.

## 4. Materials

### 4.1. Equipment

- 8-channel pipette (or 12-channel pipette), 20–200 µL volume.
- Tweezers.
- Uncapping tool (e.g., Azenta Screw Cap Remover) or flathead screwdriver, or other mechanism to facilitate unscrewing tubes.
- Label printer for duplicating rack barcode information (or generating barcode information if case racks without imprinted barcodes are used).

### 4.2. Consumables

- Nitrile or vinyl protective gloves.
- Permanent marker.
- 10 mL reagent reservoirs (“long containers”) designed for repeated filling of 8- (or 12-) channel pipettes.
- 1 mL screw-top tubes, slender enough for 8-channel pipette use (96-format) and suitable for cold storage. Tubes certified for use at freezing temperatures with external thread recommended (e.g., Azenta FluidX Next-Gen 1.0 mL jacket tube, 68-1003-11).
- 96-format tube racks (i.e., 8 × 12), with imprinted, human- (and possibly also machine-) readable barcodes, with a lockable lid (e.g., Azenta FluidX Next-Gen high-rise rack with standard lid lock, 66-51020) for ethanol-filled tubes.
- Additional 96-format tube racks for placing unused ethanol-filled tubes (see Detailed Procedure below).
- Biohazard container for the 96-well plates after mf transfer to the tubes.
- Biohazard container for used pipette tips.
- Racked pipette tips, 200 µL (96-format, i.e., 12 columns and 8 rows).

### 4.3. Solutions

- Ethanol, molecular grade, pure.

NB: ethanol with a lower concentration (e.g., 96%) can be used, but the volume of ethanol in the tubes needs to be adjusted to ensure that, following addition of the saline containing the mf, the final ethanol concentration is not below 70% and is ideally at 75–80%. An alternative storage medium, such as RNAlater^®^ (Invitrogen, Thermo Fisher Scientific, Waltham, MA, USA), can be used.

- 0.9% NaCl solution (approximately 10 mL per 96-well plate).

### 4.4. Counted Microfilariae and Associated Metadata

- 96-well plates with the skin snips and mf in 0.9% saline, after the mf have been counted and the designated staff has released the plate for mf transfer.

NB: the transfer of the mf should occur as soon as possible.

- Photocopy of completed Source Record Forms of a quality that ensures that all writing is readable (blank forms for studies that use two or four skin snips are available in the Supplemental File).

## 5. Detailed Procedure

### 5.1. Preparatory Work

Steps 1 and 2 can be performed by staff at the site where the samples are collected or by staff of the mf receiving laboratory that provides the relevant material.

1. Pre-fill tubes with 500–600 μL ethanol (≥99.5%). Ensure caps are tight to reduce evaporation and reduce the risk of spilling.
2. For each rack, print 2× labels with a unique alpha-numeric code. If the rack already has an imprinted code (i.e., pressed into the plastic frame), use the same alpha-numeric barcode as on the rack. Compare the printed barcode to the one imprinted on the rack to ensure they are identical and print new labels if they do not correspond. Stick one label on the rack itself (i.e., not on the lid). The other should be stuck on the outside of the rack such that it is easily detachable (it will be affixed to the Source Record Form in step 8). This step ensures that the label on the rack and the label on the Source Record Form will be identical in the case of a typo in the printed rack label relative to the imprinted rack barcode.
3. Fill the long containers (reagent reservoirs) with NaCl (0.9%).
4. Set the 8-channel (or 12-channel) pipette to 100 μL.
5. Match the photocopies of the completed Source Record Form and the 96-well plates with the mf via the Plate Identifier on the top left corner of the Source Record Form.

### 5.2. Transfer of Microfilariae

1. Review the photocopy of the completed Source Record Form to identify any wells that have mf (Figure 1a).
2. Align the first 96-well plate with the mf to be transferred and the rack with 96 tubes into which the mf will be transferred. Unless the rack positions are labelled, a standard approach for ensuring that the racks are facing the same way across institutions working on the same project is important. With the brand of racks used in our study, the alpha-numeric, pre-printed barcode were oriented on the left side, while the pre-printed labels are on the front facing the laboratory staff. This ensures that the material in, e.g., plate well A1 (corresponding to the counts and participant code on the Source Record Form for well A1) is transferred to the first tube A1 in the upper left of the rack. This is key to eliminating the need for labelling individual tubes (Figure 1b).
3. Remove one of the detachable barcode labels from the rack and stick it to the Source Record Form copy.
4. Unlock the rack lid and place it face up on the counter.
5. Remove those tubes in the rack that correspond to positions in the 96-well plate with 0 mf and place them into empty racks for later use (Figure 1c).
  NB: This may result in entire columns or rows without tubes since the position of the tubes remaining in the rack must correspond exactly to the position of wells with mf in the 96-well plate. While this step increases workload for laboratory staff during mf transfer, it reduces waste of ethanol-filled tubes, shipping weight, and time required for filling tubes with ethanol for future mf transfers.
  NB: for the brand used in our study, the orange capping tool can be used to push tubes out from the bottom of the rack if needed.
6. Line up the 96-pipette tip rack with the 96-well plate and the 96-well tube rack (Figure 1b).
7. Remove those pipette tips that correspond to positions in the first column of wells of the 96 well plate without mf and place them into the tray (they will be later placed into an empty 96-pipette rack) (Figure 1b).
8. Starting with the wells in column 1, corresponding to wells A1–A8 in the 96-well plate (or the first column of wells in which at least one well includes mf), follow the procedure given below:
  a. Remove the screw tops of the tubes (using the orange capping tool, flathead screwdriver, or by hand) and place them in the rack lid.
  b. Equip the pipette with tips for each of the positions corresponding to wells containing mf.
  c. Pipette the contents of the wells gently up and down three or four times to ensure that mf are pipetted up.
    NB: It is important that this step is GENTLE. Sudden or jerky pipette plunger action will increase the risk of mf being sucked into the pipette channels, contaminating the pipette and leading to loss of mf.
    NB: Take care NOT to include the skin snip in the well. The pipette tip orifice should be too small to allow accidental pipetting of the skin snip, but attention needs to be paid to this and the fact that the skin snip may temporarily block the orifice.
  d. Transfer the contents into the corresponding tubes in the rack.
  e. Set the pipette volume to 50 µL.
  f. Pipette up 50 µL NaCl from the long container.
  g. Transfer the 50 µL NaCl into the wells from which the mf were just transferred.
  h. Pipette up and down gently three or four times to rinse the wells—following the notes above.
  i. Transfer the 50 µL to the corresponding tubes in the rack (final ethanol concentration in the tubes ≥74%).
  j. Inspect the wells to confirm that the skin biopsy is still in there. For any well without a visible skin biopsy inspect the corresponding tube in the rack for the skin snip and remove with tweezers if necessary.
  k. Eject the pipette tips into the biowaste container.
  l. Re-cap the tubes in the column, ensuring that each tube is firmly closed.
9. Repeat this step for all non-empty columns 2–12.
10. Replace the lid on the rack and lock it.
11. Discard the 96-well plate into the designated biowaste container.

### 5.3. Storage of Consumables for Re-Use

1. Place the ethanol-tubes not needed for preservation of mf (because their position corresponded to a well without mf) into EMPTY tube racks. Once all positions in the rack have been filled, the tube rack can be used for transfer of mf as per Section 5.1.
2. Place all pipette tips not used (because their position corresponded to a well without mf) into an empty pipette tip rack. Once all positions in the rack have been filled, the rack can be used for future transfer of mf as per Section 5.1.

## 6. Methods for Comparison of Suitability of Microfilariae Samples Transferred without and with Our New Procedure for Genetic Analysis

### 6.1. Picking of Microfilariae

The contents of each tube with mf were tipped into a glass cavity slide. Each mf was picked using an eyelash, to which the mf adheres, under a dissecting microscope and transferred individually into 20 µL lysis buffer. Care was taken to avoid cell debris. The eyelash was washed in water between picking of mf from different tubes. NB: mf can also be picked using a pipette to bring up 1 µL of solution with a single mf and ejecting the solution into the lysis buffer tube. To test for potential cross-contamination during mf picking, storage medium without mf could be placed into a glass cavity slide, “picked” using the eyelash or pipette approach, and transferred into lysis buffer as a negative control for downstream molecular work.

For the tubes received from South Sudan, we picked all mf that we could find from each tube. Note that only a subsample of the South Sudan mf were counted, and a smaller subsample transferred to ethanol for storage and transport. In addition, because the DRC trials are ongoing at the time of this manuscript, we have picked mf only from participants that had counts of at least 50, and picked up to 48 mf per participant (which fills half of a 96-well plate and is thus a convenient stopping point for quantitative PCR (qPCR), sequencing, or other DNA-based analysis). We thus cannot directly compare the number of mf successfully transferred between studies.

### 6.2. DNA Extraction, Amplification, and Sequencing

To confirm that the new procedure was effective at preserving mf for genetic analysis and to compare this effectiveness with the transfer method used for the mf from South Sudan, we performed qPCR on single mf as described below.

#### 6.2.1. DNA Extraction

1. Lysis buffer was prepared using 10 mM Tris-HCl pH 8.0, 1 mM EDTA pH 8.0, and 1% Tween^®^20 (Sigma-Aldrich, Burlington, MA, USA), with 300 µg/mL proteinase K added just prior to use. Lysis buffer can be stored at 8 °C for no more than 24 h before use.
2. Wells each containing a single mf in lysis buffer were incubated at 55 °C for 2 h, followed by heat inactivating the solution at 80 °C or 85 °C for 20 min.

NB: the mf for which the qPCR results are presented were picked by the same person to minimize any differences in results due to sample handling.

#### 6.2.2. Detection of DNA in the Crude Lysate Using qPCR

1. Primers were used to target a 67 bp region of the mitochondrial genome [22]: forward primer, SP-Ov-mt-10062 (5′-ttg att caa tat cag gga cgt a-3′); reverse primer, ASP-Ov-mt-10062 (5′-att ggt gac caa taa cct tca-3′). As a positive control, an oligonucleotide of the mitochondrial (mt) target sequence was synthesized (5′-ttg att caa tat cag gga cgt ata ttt cgt caa tct gag ttg act ttg aag gtt att ggt cac caa t-3′). For a 66 bp region of the nuclear genome: SP_Ov_OM4_4192352 (5′-tta gag gcc ctt tcg cag t-3′) and ASP_Ov_OM4_4192352 (5′-cag cac tga tcc cgg taa at-3′) with corresponding oligonucleotide as a positive control (5′-tta gag gcc ctt tcg cag tcc ttt tag tta cat tct tca aca gtg aat tta ccg gga tca gtg ctg-3′).
2. The qPCR reactions were performed with a final volume of 10 µL containing 5 µL of SsoAdvanced Universal SYBR Green master mix (Bio-Rad Laboratories, South Granville, NSW, Australia), 2 µL of nuclease free water, 0.5 µL each of 10 µM forward and reverse primers, and 2 µL of 1:5 diluted microfilarial lysates.
3. In each qPCR run, water was used as a negative control, and the synthesized oligonucleotide was used as a standard. Each of the diluted lysates was run in duplicate, and the standards in triplicate.
4. qPCR assays were run on a CFX Real-Time System (Bio-Rad Laboratories, Hercules, CA, USA), with an initial denaturing step of 3 min at 98 °C followed by 40 cycles of (98 °C 10 s, 54 °C 15 s, 72 °C 15 s, read plate) including melt curve analysis at 65 °C to 95 °C for 5 s with an increment of 0.2 °C for 5 s.
5. Overall statistics for the qPCR runs were assessed using the CFX Maestro Software (Bio-Rad Laboratories). Cycle quantification (C_q_) values for each sample were determined as positive for the mitochondrial genome if the C_q_ < 30 and for the nuclear genome C_q_ < 33 [37,38].

## 7. Results

### 7.1. Sample Receipt and Tube Identification and Assignment to Metadata

All sample tubes from both the epidemiological study in South Sudan and the clinical studies in DRC were received at La Trobe University in good condition, with no leakage of ethanol.

Reading the handwritten labels on the individual tubes and linking those to the relevant metadata from South Sudan was challenging in some cases. In contrast, the samples received from DRC based on our new procedures were easier to sort and took less time to link the metadata on the photocopied Source Record Forms to a particular tube. Given that funding for sequencing is finite, this made it easier to identify which mf to target for genetic analysis depending on study objectives.

### 7.2. qPCR Assessment of DNA Extractions

**Figure 2:**
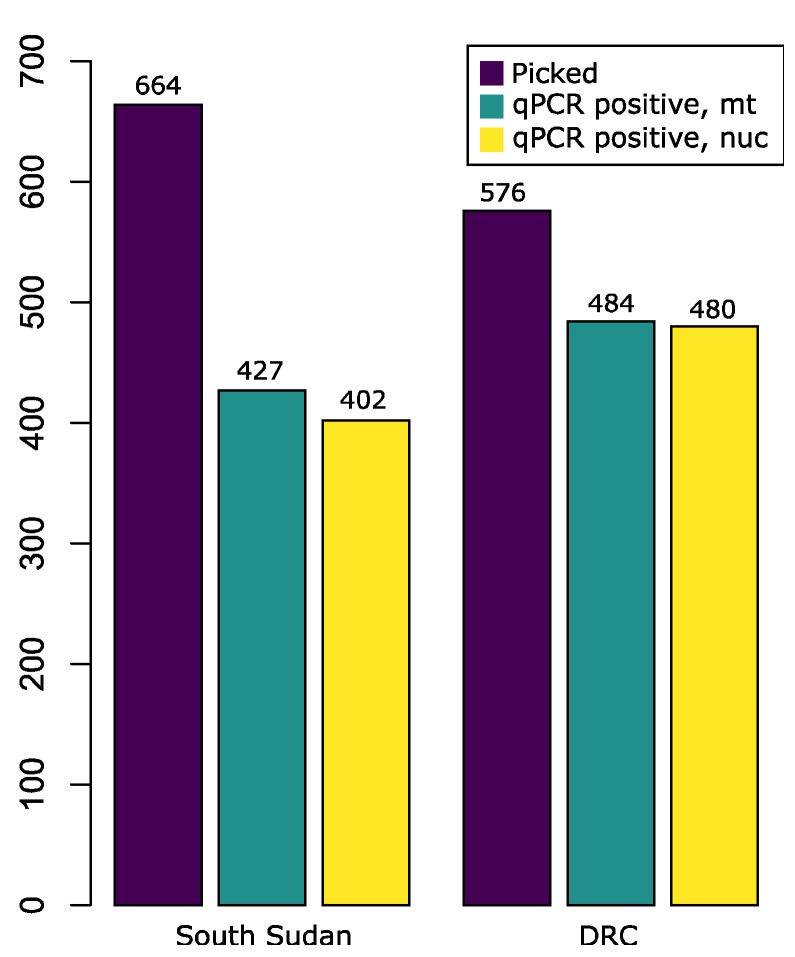
Comparison of positive qPCR of a 67 bp mitochondrial target (C_q_ < 30) and 66 bp nuclear target (C_q_ < 33) of *Onchocerca volvulus* from South Sudan (2018) and the Democratic Republic of Congo (2021). The total number of microfilariae picked and analyzed is compared to the number that successfully passed detection standards for mitochondrial (mt) DNA and nuclear (nuc) DNA.

Of the mf picked, approximately 600 mf from each country were picked by one person and used for comparison using qPCR assays. Mf were picked from 29 samples (i.e., two skin snips obtained at the same time point) from South Sudan and 7 samples (i.e., four skin snips obtained at the same time point) from the DRC. Of the mf from South Sudan, 35.7% failed the mt qPCR assay and 39.5% failed the nuclear assay, while only 16.0% and 16.7% of the mf from the DRC failed the mt and nuclear assay, respectively (Figure 2). The difference in the proportion of successful DNA extractions for either mitochondrial or nuclear markers between the two samples from the two countries was significant at *p* < 0.0001 based on a two-sample test with continuity correction performed in R v.4.2.1 [39].

## 8. Discussion

We have presented a procedure designed to maximize transfer of mf that have emerged from skin snips after microscopic examination for downstream genetic or other analytic work. As an example of such downstream work, we have used whole-genome amplification of mf followed by next-generation sequencing to estimate the minimum number of reproductively active female worms in a person [22]. Here, we report higher retention of mf and higher DNA quality when the new procedure was used for mf transfer and storage.

There are a number of factors that could contribute to the differences observed in DNA extraction success. One factor could be differences in the time between when the skin snips were obtained and their transfer into ethanol (the time for incubation in physiological saline at ambient temperature is at least 8 h and frequently overnight to allow mf to emerge from the skin, time for microscopic determination of the number of mf that emerged, time between microscopy, and mf transfer into ethanol). If the mf die during that period, the DNA may begin to degrade prior to transfer into ethanol. In addition, the mf from South Sudan were stored at a lower concentration of ethanol than those from DRC (64% vs. ≥74%). While long-term storage and differences in ethanol concentration can affect the fragment length of DNA in preserved samples [40], researchers report sequencing amplicons up to ~200 bp long from invertebrate samples preserved in 70% ethanol for up to 12 years [41]. The primers used for qPCR for this experiment intentionally targeted very small fragments of DNA (66–67 bp) to detect presence/absence of mitochondrial versus nuclear genomes, and are thus less likely to be impacted by the variation in storage conditions. In addition, the development of this procedure was motivated by high rates of whole-genome amplification failure of mf from South Sudan performed less than one year post-sampling, in 2021 (some of these data were published in [22]). Therefore, we do not think that the difference in duration of storage in ethanol before the DNA extraction (around 4 years for the samples from South Sudan collected in 2018 and around 1 year for the samples from DRC collected in 2021) is likely to contribute to the observed difference in qPCR failure rate.

The studies for which we developed the procedure are large-scale studies (a recruitment goal of 12,500 participants from whom skin snips would be obtained once and several hundred participants from whom skin snips would be obtained seven times). For small-scale drug efficacy trials or epidemiological studies identifying few mf-containing skin snips, the elements of our procedure, which we strongly recommend adopting, are the use of an inverted microscope to allow counting mf directly in the 96-well plate, washing the 96-plate wells (and pipette) with saline to increase the number of mf transferred into storage media (steps 13f–i), ensuring a final ethanol concentration of at least 75–80%, and using screw-cap tubes for transport. Whenever possible, the use of printer-generated labels should be considered to ensure readability of tube identifiers, particularly in the context of international collaborations and no international standard for handwritten numbers.

## 9. Conclusions

Nearly all onchocerciasis meso- and hyperendemic areas have been undergoing at least annual MDAi for >20 years (and in some countries additional interventions) that has substantially reduced morbidity as well as *O. volvulus* transmission and thus prevalence and intensity of infection [1,11,42,43,44]. This means that the number of individuals with onchocercal nodules containing live macrofilariae, the number of individuals with mf in the skin, and the level of mf in their skin has decreased significantly. Given the effort required to collect mf via skin snips, mf have always been a precious resource, but this resource is becoming rarer and thus even more precious as progress towards elimination of *O. volvulus* transmission advances. Consequently, preserving as many mf from skin snips as possible becomes increasingly important for development of tools for national programs and research on drug efficacy and safety.

While the results we present are not based on a ‘randomized, parallel group, operator-controlled’ trial comparing our new procedure with other procedures, the objective and subjective data we present, together with our experience with mf received from many other sources, suggest that the new procedure increases the percentage of mf in skin snips suitable for genetic analysis of single mf and reliably linked to the metadata.

## Data Availability

Not applicable.

## Supplementary Materials

The following supporting information can be downloaded at: https://www.mdpi.com/article/10.3390/tropicalmed8090445/s1, Supplemental File: Source Record Forms (four skin snips and two skin snips).

## Open Access Statement

In accordance with the WHO’s open-access publication policy for all work funded by the WHO or authored/co-authored by WHO staff members, the WHO retains the copyright of this publication through a Creative Commons Attribution IGO license (http://creativecommons.org/licenses/by/3.0/igo/legalcode accessed on 1 August 2023), which permits unrestricted use, distribution, and reproduction in any medium provided the original work is properly cited.
