# Supplementary material for: Procedure for Handling and Storage of Onchocerca volvulus Microfilariae Obtained from Skin Snips for Downstream Genetic Work
Source: Trop Med Infect Dis. 2023 Sep 12;8(9):445. doi: 10.3390/tropicalmed8090445 (PMC10536066; doi:10.3390/tropicalmed8090445)
Supplement: Supplementary file 1 [file tropicalmed-08-00445-s001.zip › tropicalmed-2583067-supplementary.pdf]

Supplemental File: Procedure for handling and storage of *Onchocerca volvulus* microfilariae obtained from skin snips for downstream genetic work

Shannon M. Hedtke [1,*], Anusha Kode [1], Tony Ukety [2], Jöel Lonema Mande [2], Germain Masikini Abhafule [2], Anirwoth Raciu [2], Claude Baga Uvon [2], Stephen R. Jada [3], An Hotterbeekx [4], Joseph Nelson Siewe Fodjo [4], Makedonka Mitreva [5], Wilson Sebit[6], Robert Colebunders [4], Warwick N. Grant [1] and Annette C. Kuesel [7]

[1] Department of Environment and Genetics, La Trobe University, Bundoora, Victoria, Australia; S.Hedtke@latrobe.edu.au (S.M.H.), A.Kode@latrobe.edu.au (A.K.), W.Grant@latrobe.edu.au (W.N.G.)
[2] Centre de Recherche en Maladies Tropicales (CRMT), Rethy, Ituri, Democratic Republic of the Congo tony.ukety@gmail.com (T.U.), joel.mande6@gmail.com (J.L.M.)
[3] Amref South Sudan, P.O. 30125 Juba, Republic of South Sudan; Stephen.Jada@amref.org (S.R.J.)
[4] Global Health Institute, University of Antwerp, Antwerp, Belgium; an.hotterbeekx@uantwerpen.be (A.H.); JosephNelson.SieweFodjo@uantwerpen.be (J.N.S.F.); robert.colebunders@uantwerpen.be (R.C.)
[5] Department of Medicine, Washington University in St. Louis and McDonnell Genome Institute, St. Louis, Missouri, U.S.A.; mmitreva@wustl.edu (M.Mi.)
[6] National Public Health Laboratory, Juba, Republic of South Sudan; Wilsonladu0@gmail.com (W.S.)
[7] UNICEF/UNDP/World Bank/World Health Organization Special Programme for Research and Training in Tropical Diseases (TDR), World Health Organization, Geneva, Switzerland (retired as of March 2023); kuesela@who.int (A.C.K.)
* Correspondence: S.Hedtke@latrobe.edu.au (S.M.H.)

Source Record Forms for :

(1) 4 skin snips taken per participant : right (R) and left (L) iliac crest (IC), right and left calf;

(2) 2 skin snips taken per participant : right (R) and left (L) iliac crest (IC).

Source Record Form: Skin Microfilariae

Health District ________________________ Village ___________________ Visit _____________

Snip Date ___/___/20__ FIRST snip time: ____:____LAST snip time: ____:____ Snipping technician ___________

FIRST Count Date ___/___/20__ FIRST count time: _____:_____ Reading technician ____________

Plate		1	2	3	4	5	6	7	8	9	10	11	12
Participant Code													
A R IC	Wt												
	Count												
B L IC	Wt												
	Count												
C R Calf	Wt												
	Count												
D L Calf	Wt												
	Count												
Mean Density													
Participant Code													
E R IC	Wt												
	Count												
F L IC	Wt												
	Count												
G R Calf	Wt												
	Count												
H L Calf	Wt												
	Count												
Mean Density													

Health district ________________________ **Village** ____________________ **Visit** ______________________

Snip Date ___/___/20__ **FIRST snip time:** ____:____**LAST snip time:** ____:____ **Snipping technician** ___________

FIRST Count Date ___/___/20__ **FIRST count time:** _____:_____ **Reading technician** ____________

Plate		1	2	3	4	5	6	7	8	9	10	11	12
Participant Code*													
A R IC	Wt												
	Count												
B L IC	Wt												
	Count												
Mean Density													
Participant Code													
C L IC	Wt												
	Count												
D L IC	Wt												
	Count												
Mean Density													
Participant Code													
E R IC	Wt												
	Count												
F L IC	Wt												
	Count												
Mean Density													
Participant Code													
G L IC	Wt												
	Count												
H L IC	Wt												
	Count												
